# Prediction of EGFR Mutation Status Based on ^18^F-FDG PET/CT Imaging Using Deep Learning-Based Model in Lung Adenocarcinoma

**DOI:** 10.3389/fonc.2021.709137

**Published:** 2021-07-22

**Authors:** Guotao Yin, Ziyang Wang, Yingchao Song, Xiaofeng Li, Yiwen Chen, Lei Zhu, Qian Su, Dong Dai, Wengui Xu

**Affiliations:** ^1^Department of Molecular Imaging and Nuclear Medicine, Tianjin Medical University Cancer Institute and Hospital, National Clinical Research Center for Cancer, Tianjin Key Laboratory of Cancer Prevention and Therapy, Tianjin’s Clinical Research Center for China, Tianjin, China; ^2^School of Medical Imaging and Tianjin Key Laboratory of Functional Imaging, Tianjin Medical University, Tianjin, China

**Keywords:** adenocarcinoma of lung, fluorodeoxyglucose F18, positron emission tomography computed tomography, deep learning, epidermal growth factor receptor

## Abstract

**Objective:**

The purpose of this study was to develop a deep learning-based system to automatically predict epidermal growth factor receptor (EGFR) mutant lung adenocarcinoma in ^18^F-fluorodeoxyglucose (FDG) positron emission tomography/computed tomography (PET/CT).

**Methods:**

Three hundred and one lung adenocarcinoma patients with EGFR mutation status were enrolled in this study. Two deep learning models (SE_CT_ and SE_PET_) were developed with Squeeze-and-Excitation Residual Network (SE-ResNet) module for the prediction of EGFR mutation with CT and PET images, respectively. The deep learning models were trained with a training data set of 198 patients and tested with a testing data set of 103 patients. Stacked generalization was used to integrate the results of SE_CT_ and SE_PET_.

**Results:**

The AUCs of the SE_CT_ and SE_PET_ were 0.72 (95% CI, 0.62–0.80) and 0.74 (95% CI, 0.65–0.82) in the testing data set, respectively. After integrating SE_CT_ and SE_PET_ with stacked generalization, the AUC was further improved to 0.84 (95% CI, 0.75–0.90), significantly higher than SE_CT_ (p<0.05).

**Conclusion:**

The stacking model based on ^18^F-FDG PET/CT images is capable to predict EGFR mutation status of patients with lung adenocarcinoma automatically and non-invasively. The proposed model in this study showed the potential to help clinicians identify suitable advanced patients with lung adenocarcinoma for EGFR‐targeted therapy.

## Introduction

Lung cancer is one of the leading causes of cancer-related death around the world (1, 2). Non-small cell lung cancer (NSCLC) account for more than 80% of the total number of lung cancer cases, among which the adenocarcinoma is the most common histological subtype (3). As the development of the molecular biology, the discovery of epidermal growth factor receptor (EGFR) and the emergence of small molecular tyrosine kinase inhibitors (TKIs) targeting EGFR mutations, such as gefitinib and erlotinib, have revolutionized the treatment of advanced NSCLC (4). Compared with traditional chemotherapy, EGFR-TKI has fewer side effects and has been proven to more significantly improve the prognosis of NSCLC patients with EGFR mutations (5). However, for the patients without EGFR mutations, EGFR-TKI not only has no effect, but may cause worse prognosis than platinum‐based chemotherapy (6), suggesting the importance of EGFR mutation detection.

Mutation profiling of the biopsies from advanced patients or surgically removed samples from early-stage patients have become the golden standard of mutation detection. However, difficulty of accessing sufficient tumor tissue samples and poor DNA quality partly limit the application of mutation profiling (7). Furthermore, because of the poor physical fitness, invasive examinations, such as biopsy, were not suitable for advanced patients with lung cancer. Therefore, there is an urgent need for a non-invasive way to predict EGFR mutations.

^18^F-FDG PET/CT is a widely used imaging modality in clinical practice and has been proven to play an important role in the diagnosis, staging, and prognostic evaluation of lung cancer (8–10). Recent researches have shown that EGFR signaling regulates the glucose metabolic pathway, which could be reflected by the uptake of ^18^F-FDG, indicating the potential of predicting EGFR mutation status by ^18^F-FDG PET images (11, 12). Some researchers also found that the radiomic features of PET images were associated to EGFR mutation (13). Besides, previous study has also demonstrated that radiomic features derived from CT images also showed predicting value to EGFR mutation status (14). However, the extraction of radiomic features required the precise delineation of the lesions, which is time-consuming (15). Also, the radiomic features may be affected by the imaging parameters and delineation accuracy, causing poor repeatability of some of them (16).

As the continuous development of computer technology, one of the deep learning algorithms, convolutional neural networks (CNNs), has shown a promising performance in lesion detection, segmentation, and classification (17–19). Compared with the feature engineering-based radiomic methods, CNNs do not require the precise delineation of tumor (20). Moreover, CNNs could automatically learn the features, which were more specific to the clinical outcome (19). Nowadays, some researchers focused on predicting EGFR mutation status with deep learning models. Zhao et al. constructed a DenseNet on CT images to predict EGFR mutation, and the AUC of the model was 0.75 (21). Wang et al. further improved the predictive performance by training models with contrast-enhanced CT images (19). Mu et al. built a deep learning model to predict EGFR mutation by registering and fusing PET/CT images at the image level, and the results showed that the AUC of model trained with fused images has been significantly improved to 0.85 than trained with PET or CT image alone (22). These suggest that integrating multiple information could improve the prediction accuracy of the model to a certain extent. In the clinical practice, the pulmonary function of patients with advanced lung cancer was relatively poor, and the amplitude of respiratory movement was larger than other early-stage patients. It may be more challenging in registering PET and CT imaging in this situation (23).

Considering the abovementioned situation, we develop a deep learning-based model in ^18^F-FDG PET/CT images to predict the EGFR mutant status in patients with pulmonary adenocarcinoma. We first separately built and trained the deep learning models based on CT and PET images, and then used another model to synthesize the predictive results of the CT model and the PET model to give the final prediction of EGFR mutation. The proposed deep learning-based model could help clinicians identify suitable advanced patients with lung adenocarcinoma for EGFR-targeted therapy, facilitating implementation of precise medicine with an efficient and convenient way.

## Materials and Methods

### Creation of Data Set

**Figure 1:**
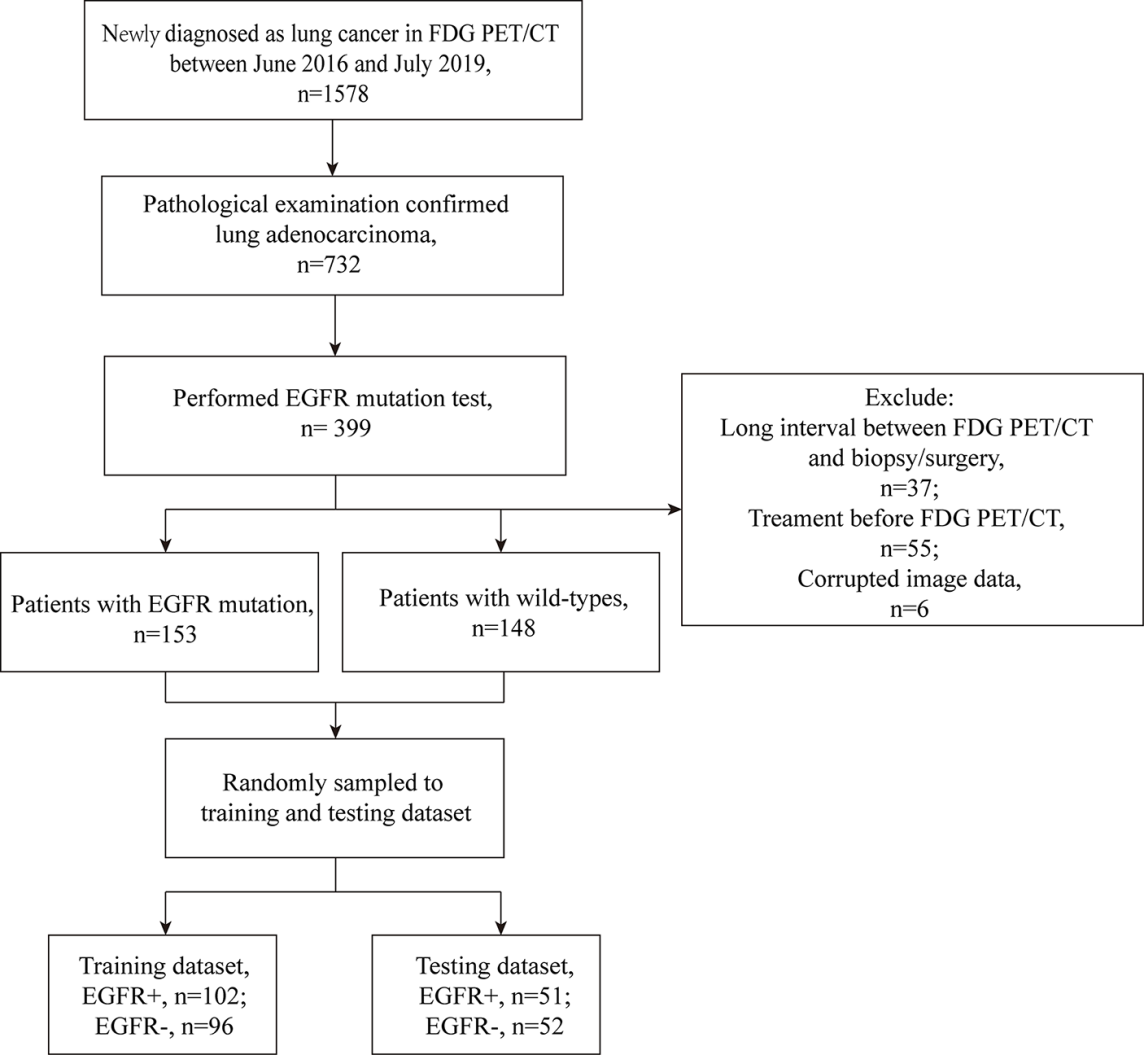
The process of the data set establishment. Long interval: exceeding 2 weeks. Corrupted image data: the CT or PET data that cannot open.

This retrospective study used the local data collected in Tianjin Medical University Cancer Hospital. Patients between June 2016 and July 2019 who meet the following inclusion criteria were included in this study. 1) patients performed ^18^F-FDG PET/CT imaging before surgery or aspiration biopsy and the image data could be obtained; 2) the pathological reports of the specimens confirmed primary pulmonary adenocarcinoma; 3) the specimens obtained by surgical resection or aspiration biopsy have been tested for EGFR mutation. Patients were excluded if 1) neo-adjuvant chemotherapy/radiotherapy was received before ^18^F-FDG PET/CT imaging; 2) the duration between surgery/biopsy and ^18^F-FDG PET/CT imaging exceed 2 weeks. Finally, 301 patients were included in this study, and patients were split into training and testing data set. **Figure 1** showed the process of the creation of data set. All procedures in studies involving human participants were conducted in accordance with the 1964 Helsinki declaration and its later amendments or comparable ethical standards.

### EGFR Mutation Profiling

EGFR mutations were identified on exons 18, 19, 20, and 21, which were the main drug target-associated mutations. For the surgical resected specimens, the EGFR mutations were examined using quantitative real-time polymerase chain reaction. For the aspiration biopsied specimens, the EGFR mutations were examined by high-performance capillary electrophoresis. All specimens were taken from the primary lung tumor masses. If the mutation of any of the above exons were detected, the lesion was defined as EGFR-mutant; otherwise, the lesion was defined as EGFR-wild type.

### ^18^F-FDG PET/CT Procedure

Images were obtained using GE Discovery Elite PET/CT scanner (GE Medical Systems). Patients fasted for approximately 6 h with a serum glucose level <11.1mmol/L before PET/CT imaging. Images were started to acquire 50 to 60 min after injection of 4.2 MBq/kg ^18^F-FDG. A spiral CT scan (80 mAs, 120 kVp, 5-mm slice thickness) was first acquired for precise anatomical localization and attenuation correction, and a PET emission scan (3D mode) was subsequently followed from the distal femur to the top of the skull. PET images were reconstructed using iterative algorithms ordered-subset expectation maximization (OSEM) to a final pixel size of 5.3 × 5.3 × 2.5 mm. A 6-mm full-width at half maximum Gaussian filter was applied after the reconstruction.

### Data Preprocessing

The spacing of ^18^F-FDG PET and CT images were first resampled to 1×1×1 mm^3^ by third-order spline interpolation to avoid the image distortion. Then, the regions of interest (ROIs) with size of 64 mm × 64 mm, which centered on primary lung tumor were manually selected for PET and CT images by two radiologists with 3- and 4-year experience in ^18^F-FDG PET/CT diagnosis using medical image processing software 3D Slicer (version 4.10.2), and subsequently confirmed by a 10-year experienced nuclear medicine physician. To reduce the influence of the difference between the middle level slices and the peripheral level slices on the performance of models, only 80% of all tumor slices centered on the largest slice were selected as ROIs. After the segmentation, the ROIs were exported as NII format for further analysis. Before feed into the models, the ROIs were normalized according to the following methods: the CT ROIs were converted into Hounsfield units with the range of −1,000 to 200, and the values were transformed to [−1, 1); the PET ROIs were converted into standard uptake values with the range of 0 to 40 and transformed to [−1, 1). The ROIs were labeled as EGFR mutant (Mut) or wild type (WT) according to the corresponding EGFR mutation testing report. No image augmentation was used in this study.

### Model Architecture

To use the information in the limited data more effectively, we adopted the powerful deep convolutional neural network structure SE-ResNet module (24), which integrates residual learning for feature reuse and squeeze-and-excitation operations for adaptive feature recalibration, for PET and CT images, respectively (25). SE-ResNets have achieved great success in natural images recognition tasks. In the SE-ResNet module, the shortcut connection could enhance information flow over feature propagation and mitigate the phenomenon of vanishing/exploding gradients and network degradation in deeper networks (25). Also, the SE block could selectively emphasize informative channel features and suppress less useful ones by feature recalibration process. The SE-residual module can be formulated as below [The following formula and explanation refer to (24–27)]:

$$X^{res}=F_{res}(X)$$

Here X represents the input feature. *F_res_* consisted of three consecutive convolution-batch normalization-leaky ReLU layers. *X^res^* is the residual feature which is calculated from X by *F_res_* In the first squeezing step, the channel-wised parameter **s** = [*s*_1_, *s*_2_, … , *s_c_*] ∈ ℝ^C^ is generated by squeezing $X^{res}=\left[x_{1}^{res},x_{2}^{res},\ldots ,x_{c}^{res}\right]\in {\text{ℝ}}^{H\times W}$ through plane dimensions *H×W*, where

$$s_{c}=\frac{1}{H\times W}\sum_{i=1}^{H}\sum_{j=1}^{W}x_{c}^{res}(i,j)$$

*C* represented the number of channels of the residual feature.

To make use of the information aggregated in the squeeze operation, the second step, which aims to fully capture channel-wise dependencies, is adopted. Two fully connected layers were used to automatically identify the importance of different channels. The output of these fully connected layers can be defined as

$$\tilde{S}=\sigma (W_{2}\delta (W_{1}s))$$

Here *δ* is the Leaky ReLU function with negative slope = 0.5, *σ* is the Sigmoid function, $W_{1}\in {\mathbb{R}}^{\frac{C}{r}\times C}$, and $W_{2}\in {\mathbb{R}}^{\frac{C}{r}\times C}$ is the weights of two fully connected layers. The reduction ratio *r* is set to 8 to reduce the costs of computation.

The output of the last convolution layer in SE-Residual module is defined as ${\tilde{X}}^{res}=[{\tilde{X}}_{1}^{res},{\tilde{X}}_{2}^{res},\ldots ,{\tilde{X}}_{3}^{res}]$, where

$${\tilde{X}}_{c}^{res}=\tilde{s_{c}}\cdot {\tilde{X}}_{c}^{res}$$

**Figure 2:**
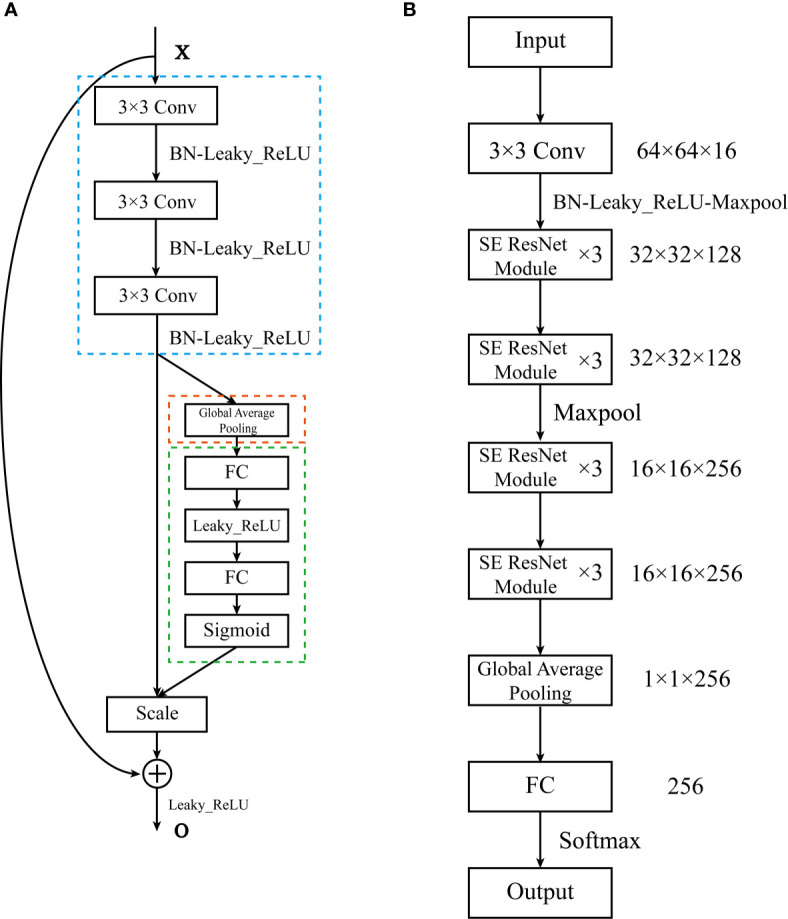
The architecture of the SE-ResNet. **(A)** The structure of the SE-Residual module. The structure in the blue dashed line is *F_res_*, the structure in the orange dashed line is the squeezing step, the structure in the green dashed line is the excitation step. **(B)** The composition of the SE-ResNet. The SE-ResNet consists of 4 basic modules. Each basic module is composed of 3 SE-Residual modules. A fully connected layer was attached to the end of the model.

Here $\tilde{s_{c}}\in \tilde{S}$ and ${\tilde{X}}_{c}^{res}$ refers to channel-wise multiplication between the feature map $X_{c}^{res}$ and the learned scale value $\tilde{s_{c}}$. The scale value $\tilde{s_{c}}$ represents the importance degree of *c*th channel. Considering the shortcut connection which could propagate gradients further by skipping one or more layers in deep nets, the final output of SE-Residual module is defined as

$$O=\delta ({\tilde{X}}^{res}+X)$$

where *δ* refers to the Leaky ReLU function with negative slope = 0.5. The basic SE-Residual module and the structure of SE_PET_ and SE_CT_ are illustrated in **Figure 2**.

Then we used stacked generalization (Stack_PET-CT_) to integrate SE_CT_ and SE_PET_ to further improve the accuracy of prediction. Stacked generalization or stacking is a model fusion method of using a high-level model to combine lower-level models to achieve greater predictive accuracy (28). The higher-level model, called “meta-classifier,” could discover the best way of how to combine the outputs of the base classifiers (29). In this study, SE_CT_ and SE_PET_ served as base classifiers. And the support vector machine (SVM) with radius-basis kernel served as the meta-classifier. We implemented the neural networks and SVM with Pytorch 1.6.0 and scikit-learn 0.23.2 based on Python 3.7.6 (30, 31).

### Model Training

For the deep learning models, the training data set was used to fit and tune models *via* fivefold cross-validation, and the testing data set was used to evaluate the predictive and generalization ability of the models. The SE_CT_ and SE_PET_ were initialized by MRSA method (32). During training, the study sampled the training data with a ratio of 1: 1 for the Mut and WT with a batch size of 128. Adam optimizer was used to update the deep learning models parameters (33). The initial learning rate was set to 5 × 10^−6^ and decayed by a factor of 1/10 at the end of epoch = 40. Weight decay of 10^−4^ was also used in the optimizer of SE_CT_ to avoid overfitting. We early stop the training after 80 epochs. The training of deep learning models was performed with an Nvidia RTX 2060 graphics processing unit (GPU).

**Figure 3:**
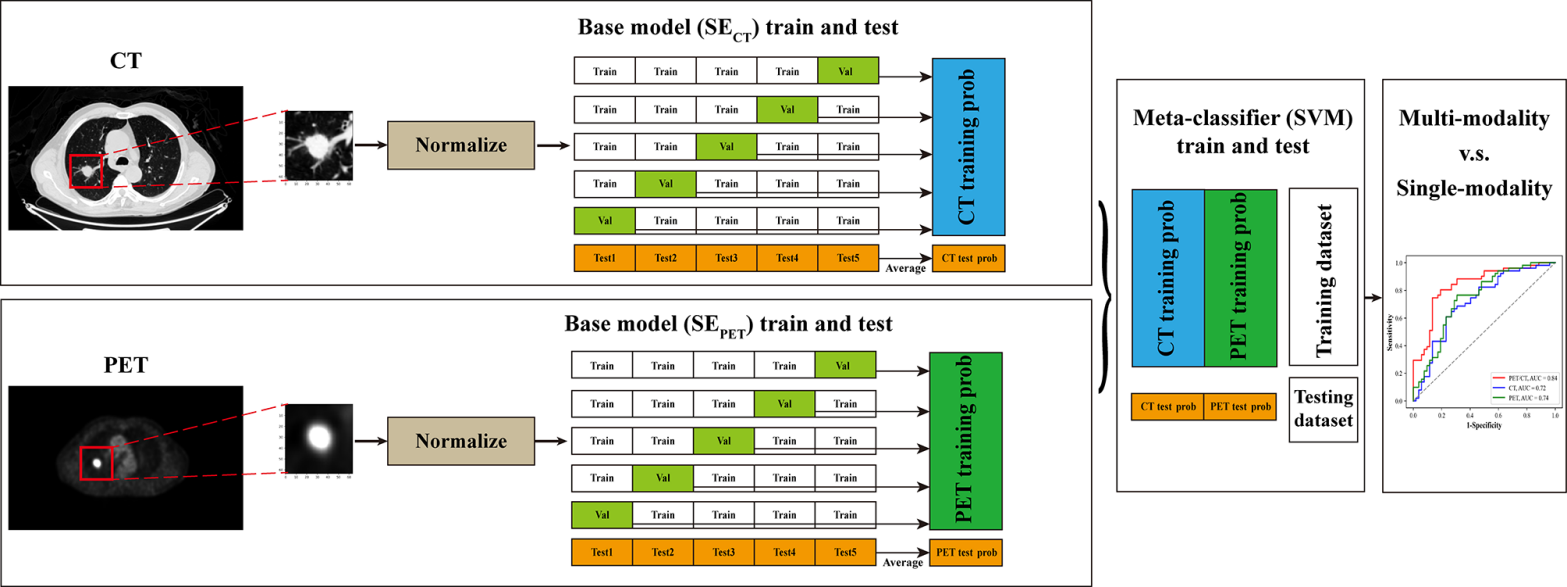
The pipeline of this study. The CT and PET images were first resampled, and the ROIs centered the primary lung tumor were manually selected and normalized. Then SE_CT_ and SE_PET_ served as base classifiers and were trained on training data set through fivefold cross-validation to get the EGFR mutation probabilities of training data set. Simultaneously, these models were tested on testing data set for five times. The predictive probabilities of SE_CT_ and SE_PET_ for training data set were combined and used for the training of SVM, which served as meta-classifier. And the five times predictive probabilities of SE_CT_ and SE_PET_ for testing data set was averaged respectively and combined for the testing of SVM. Finally, the performance of multi-modal stacking model and single-modal deep learning models was compared through ROC curve analysis.

For the Stack_PET-CT_, the meta-classifier, SVM, was trained as follows: suppose the training data set as $D_{primary}=\{(x_{n}^{CT},x_{n}^{PET},y_{n}),n=1,\ldots ,N\}$, where $x_{n}^{CT}\text{ and }x_{n}^{PET}$ are tensors representing the attribute values of the CT and PET images, and *y_n_* is the class value. Then, *D*_primary_ was randomly partitioned into five almost equal size parts *D*_1_, … , *D*_5_, and define *D_–i_* = *D_primary_* – *D_i_*, where *i*=1, …,5. *D_i_* and *D_–i_* are used as validation set and training set for the *i*th fold of the 5-fold cross-validation, respectively. The SE_CT_ and SE_PET_ are trained using instances of the training set *D_–i_* to output the hypothesis $H_{primary}^{\left(-i,CT\right)}\text{ and }H_{primary}^{\left(-i,PET\right)}$. For each pairs of instances $x_{n}^{CT}\text{ and }x_{n}^{PET}$ in *D_i_*, the validation set of the *i*th cross-validation fold, let $p_{n}^{CT}\text{ and }p_{n}^{PET}$ denote the Mut probabilities of the hypothesis $H_{primary}^{\left(-i,CT\right)}\text{ and }H_{primary}^{\left(-i,PET\right)}$ on $x_{n}^{CT}\text{ and }x_{n}^{PET}$, respectively. By processing the whole 5-fold cross-validation, the secondary training set $D_{\sec ondary}=\{(p_{n}^{CT},p_{n}^{PET}),n=1,\ldots ,N\}$ is assembled from the outputs of the two hypotheses. Then, the SVM that we call the meta-classifier is used to derive a hypothesis *H_secondary_* from the secondary training set *D_secondary_*. The development of Stack_PET-CT_ was shown in **Figure 3**. The probability of EGFR mutation at the patient level was calculated as averaging the EGFR mutation probabilities of slices that included tumor mass.

### The Interpretability of Deep Learning Models

The visualization method named Grad-CAM was used to explain the predictive process of SE_CT_ and SE_PET_ (34). The Grad-CAM algorithm could generate the attention map on the input image. The attention map can reflect the discriminative area that the deep learning models mainly focuses on in the classifying process.

### Statistical Analysis

Statistical analysis was performed using Medcalc 19.0.4 and the machine learning module scikit-learn 0.23.2 basing on Python3.7.6. The Mann-Whitney U test was used to assess the significance of the ages between Mut and WT groups. The independent-samples *t*-test was used to assess the significance of the mean value on tumor size between Mut and WT groups. The Chi-squared test was used to evaluate the difference of sex, tumor location, smoking history, and stage in all the patients. DeLong test was used to evaluate the difference of the receiver operating characteristic (ROC) curves between various models. A p-value <0.05 was treated as significant.

## Results

### Clinical Characteristic of Patients

The clinical characteristics of patients enrolled in this study were present in **Table 1**. In the training data set, 1.01% (2/198) patients had exon 18 mutation, 17.17% (34/198) patients had exon 19 mutation, 3.03% (6/198) patients had exon 20 mutation, and 30.30% (60/198) patients had exon 21 mutation. In the testing data set, 0.97% (1/103) patients had exon 18 mutation, 19.42% (20/103) patients had exon 19 mutation, 2.91% (3/103) patients had exon 20 mutation, 26.21% (27/103) had exon 21 mutations. The differences of sex and smoking history between Mut and WT were significant in both training and testing data set.

**Table 1:** Clinical characteristics of patients.

	Training data set		*p*-value	Testing data set		*p*-value
	Mut (n=102)	WT (n=96)		Mut (n=51)	WT (n=52)	
Sex			0.0091			0.0045
Male	47 (46.08)	62 (64.58)		19 (37.25)	34 (65.38)	
Female	55 (53.92)	34 (35.42)		32 (62.75)	18 (34.62)	
Age (median (range))	63 (37-75)	63.5 (28-74)	0.30	60 (43-86)	60 (47-77)	0.89
Tumor Location			0.23			0.62
Left lobes	71 (69.61)	59 (61.46)		21 (41.18)	24 (46.15)	
Right lobes	31 (30.39)	37 (38.54)		30 (58.82)	28 (53.85)	
Smoking History			0.0049			0.044
Yes	30 (29.41)	47 (48.96)		12 (23.53)	22 (42.31)	
No	72 (70.59)	49 (51.04)		39 (76.47)	30 (57.69)	
Tumor size	2.76 ± 1.00	2.97 ± 1.30	0.21	2.59 ± 0.63	2.88 ± 1.05	0.10
Stage			0.47			0.48
I	58 (56.86)	45 (46.88)		33 (64.70)	27 (51.93)	
II	11 (10.78)	14 (14.58)		7 (13.73)	8 (15.38)	
III	9 (8.82)	13 (13.54)		4 (7.84)	4 (7.69)	
IV	24 (23.54)	24 (25.00)		7 (13.73)	13 (25.00)	

Categorical variables are presented as n (%).

### The Performance of Deep Learning Models

The predictive performance of deep learning models was evaluated through the area under ROC curve (AUC), sensitivity, specificity, and accuracy. The AUC ranges from 0.5 to 1.0. The performance of model is improving as the AUC increases. Sensitivity is the numerical ratio of true EGFR mutant ones to the predicted EGFR mutant ones according to the model. It reflects the ability of find EGFR mutation. Specificity is the numerical ratio of true wild type ones to the predicted wild type ones by the model. It reflects the ability of model to identify non-EGFR mutation. Accuracy was used to evaluate the correct proportion of the model on all samples. The Stack_PET-CT_ had the highest AUC and significantly outperformed SE_CT_ and SE_PET_ in the training data set (Stack_PET-CT_ *vs*. SE_CT_: p<0.0001; Stack_PET-CT_ *vs*. SE_PET_: p<0.0001) (**Table 2**). There was the same trend in the testing data set, but the differences between Stack_PET-CT_ and SE_PET_ were not significant (Stack_PET-CT_ *vs*. SE_CT_: p=0.0056<0.05; Stack_PET-CT_ *vs*. SE_PET_: p=0.061) (**Table 3**). The Stack_PET-CT_ also had the highest specificity, accuracy, and a relatively high and stable sensitivity in both training and testing data set. There was no difference between the predictive performance of SE_CT_ and SE_PET_ in training data set (p=0.70) and testing data set (p=0.74).

**Table 2:** Predictive performance of different models in the training data set.

	AUC (95% CI)	Sensitivity (%)	Specificity (%)	Accuracy (%)
Stack_PET-CT_	**0.86 (0.80-0.91)**	71.75	**84.38**	**75.25**
SE_CT_	0.74 (0.67-0.80)	82.35	53.12	67.17
SE_PET_	0.75 (0.69-0.81)	**86.25**	56.25	72.22
Clinical model	0.64 (0.57-0.71)	65.69	62.50	60.10

The bold values represented the highest one of the evaluation indices.

**Table 3:** Predictive performance of different models in the testing data set.

	AUC (95% CI)	Sensitivity (%)	Specificity (%)	Accuracy (%)
Stack_PET-CT_	**0.84 (0.75-0.90)**	80.39	**80.77**	**73.79**
SE_CT_	0.72 (0.62-0.80)	68.63	69.23	68.93
SE_PET_	0.74 (0.65-0.82)	76.47	69.23	67.96
Clinical model	0.64 (0.54-0.73)	**86.27**	40.38	59.22

The bold values represented the highest one of the evaluation indices.

### Comparison Between the Deep Learning Models and Clinical Model

**Figure 4:**
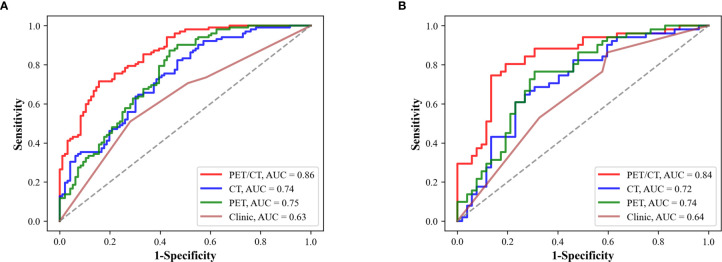
Predictive performance of SE_CT_, SE_PET_, Stack_PET-CT_, and clinical model. **(A)** The performance of different models in the training data set. **(B)** The performance of the models in the testing data set. Stack_PET-CT_ had the highest AUC in the training and testing data set.

An SVM model with linear kernel was used to build the clinical model. The clinical model included sex and smoking history, which were significantly different between Mut and WT group in training and testing data set. The Stack_PET-CT_ outperformed clinical model in both training and testing data set (Train: Stack_PET-CT_ *vs*. clinical model: p<0.0001; Test: Stack_PET-CT_ *vs*. clinical model: p=0.0022<0.05). The performance of SE_CT_ and SE_PET_ was higher than the clinical model in both training and testing data set. However, only the differences between SE_CT_ and clinical model, SE_PET_ and clinical model in training data set were significant (Train: SE_CT_ *vs*. clinical model: p=0.019<0.05; SE_PET_ *vs*. clinical model: p=0.0044<0.05; Test: SE_CT_ *vs*. clinical model: p=0.32; SE_PET_ *vs*. clinical model: p=0.13). We also build a stacking model (Stack_PET-CT-Clinical_) that combines the SE_CT_, SE_PET_, and clinical model with SVM. However, the performance of this model was not significantly improved compared with the Stack_PET-CT_ (Training AUC: 0.85, 95% CI 0.79-0.90; Testing AUC: 0.83, 95% CI 0.75-0.90). **Figure 4** shows the ROC curve of Stack_PET-CT_, SE_CT_, SE_PET_, and clinical model in the training and testing data set.

### Suspicious Area Discovered by Deep Learning Models

**Figure 5:**
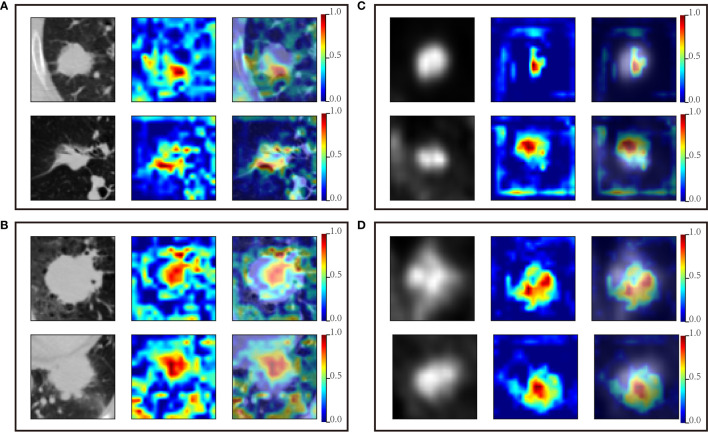
Suspicious areas generated by SE_CT_ and SE_PET_. The first column is the original PET or CT image; the second column is the attention map for classifying EGFR mutation status; the third column is the image fusing original image and the attention map. **(A)** CT images predicted as EGFR mutation by the SE_CT._ **(B)** CT images predicted as wild-type EGFR by the SE_CT_. **(C)** PET images predicted as EGFR mutation by the SE_PET_. **(D)** PET images predicted as wild-type EGFR by the SE_PET_.

**Figure 5** showed the predictive process of SE_CT_ and SE_PET_. Red area is the suspicious areas that deep learning models mainly focused on in the process of predicting EGFR mutation status. The suspicious areas were various among different tumors. In **Figure 5A**, SE_CT_ considered these tumors as EGFR mutant ones by the patterns of areas near the edge of the tumor and the ground-glass area. While in **Figure 5B**, SE_CT_ explains these tumors as wild-type ones based on the pattern of central areas. Similarly, SE_PET_ could determine whether the tumor was EGFR mutant or wild-type based on the pattern of suspicious area with high or low FDG uptake. In addition, some lung tissues in CT images also attracted the attention of SE_CT_, but the main focus was still on the tumor area.

## Discussion

For the patients with advanced pulmonary adenocarcinoma, platinum-based chemotherapy supplemented with local radiotherapy remains the major treatment. Compared with traditional treatment, molecule-targeted drugs represented by EGFR-TKI have significantly improved the prognosis of patients with advanced lung cancer. EGFR mutation status is critical to the efficacy of EGFR-TKI. In this study, we developed a stacking model based on SE-ResNet using non-invasive ^18^F-FDG PET/CT images to predict EGFR mutation status for patients with lung adenocarcinoma. After the integration of PET and CT image information with stacked generalization, the performance has been obviously improved than single modality model.

Previous studies mainly used the clinical characteristics, conventional metabolic parameters, and radiomics features of ^18^F-FDG PET/CT to predict EGFR mutation status in patients with lung cancer, such as tumor margin, CEA level, smoking history, and SUVmax (35). However, the clinical features and metabolic parameters could only reflect few information of the tumors. And the differences of conventional metabolic parameters between EGFR mutation and wild-types were controversial, leading to the unsatisfactory predictive performance (35–37). With the advent of radiomic method, the utilization of information in images has been significantly improved. Radiomic method could obtain more and quantified information of tumors by extracting features from the images. Zhang et al. combined the clinical and radiomic features with machine learning algorithms to predict EGFR mutation status, and AUC reached 0.827 (38). They also found that the radiomic features of EGFR mutation representing tumor heterogeneity were higher than wild-types, similar to the result of Zhang et al. (39). Although radiomic method has significantly improved the predictive performance, precise manual delineation of tumor required rich clinic experience, and a lot of time, which increase the pressure of radiologists.

With the emergency of deep learning algorithm, this problem has been solved to a large extent. Deep learning algorithm could predict EGFR mutations by automatically extracting and integrating features, which only requires the users to define an approximate location of tumors. It could provide more information, which was highly related to EGFR mutation than radiomic method and clinical features with an end-to-end training process (19, 21). In this study, the prediction of EGFR mutation status was mainly based on the tumor area, similar to the result of previous studies (19, 22). For CT images, because of the similar density of some tumor tissue and the lung structure, such as pulmonary blood vessels, the lung tissue surrounding the tumor also attracted the attention of the SE_CT_ to a certain extent. It may be the reason that the performance of SE_CT_ was inferior to Wang et al. model, which was trained with contrast-enhanced CT images. Nevertheless, SE_CT_ could still mainly focus on the tumor. This phenomenon was relative rare in PET images because of the obvious difference between the FDG uptake of tumor lesion and surrounding lung tissue. This may also be the reason that the performance of SE_PET_ was better than SE_CT_.

Previous studies have shown that integrating multi-modal information could significantly improve predictive performance (22, 40). Considering that the registration of PET and CT images has certain difficulties in advanced lung cancer patients with poor lung function, we performed stacked generalization to integrate the information in PET and CT images. Stacked generalization can be viewed as a means of collectively using several classifiers to estimate their own generalizing biases, and then filter out those biases (28). Traditional stacking is a model with hierarchical structures that is generally built for a same data set. Previous studies have proven that the stacking model could perform at least as well as the best based classifier included in the ensemble (41, 42). And the performance of stacking model will be gradually improved at the increase of the diversity of the based classifiers. In this study, we focused on another form to implement stacked generalization that integrate two base models trained with different data sets, which were different aspects of the same object. After integrating the information of PET and CT images in this method, the AUC was improved from 0.72 and 0.74 to 0.84, similar to the results of Mu et al, further proving that multi-modal fusion could further improve the predicting performance. This result also indicated that stacking strategy is also suitable for the combination of models built with different aspects of the same object.

There was still some limitation in our study. First, because of the random sampling error, the lesions in the training data set are mainly located in left lobes, and most of the lesions in the test data set are located in the right lobe. Nevertheless, the error will not significantly impact the performance of the deep learning models, because the deep learning model uses the local primary lung tumor images as the data, which does not contain the location information of lesions. Second, the performance of Stack_PET-CT-Clinical_ has not been further improved compared to Stack_PET-CT_. The reason is that in this strategy, a significant improvement of the meta-classifier performance requires the relatively good and consistent performance of the base models, whereas the clinical model was not as good as SE_CT_ and SE_PET_, resulting in no further improvement in the performance of Stack_PET-CT-Clinical_. Building clinical models with more and effective clinical features may solve this problem. Third, the deep learning models were trained with 2D axial images. Training the model with 3D imaging data through multi-view may further improve the predicting performance. Besides, the CT and PET images used in this study are thick-slice, and the blood supply of the tumor is not considered. Further study with thin-slice enhanced CT may further improve the performance of deep learning models. Lastly, it was a single-center study with a small sample size, which only included Asian population with a relatively high percentage of EGFR mutation. The limited sample size may be the reason of insignificant difference between the performance of clinical model and SE_CT_, SE_PET_ in testing data set. The deep learning models require larger and more diverse data set to be fine-tuned and needs to be further tested in larger cohorts. A further multi-center study with a large sample size and multiple races may improve the generalization of the model to a certain extent.

In conclusion, we developed a deep learning-based model using ^18^F-FDG PET/CT images to predict the EGFR mutation status in patients with lung adenocarcinoma. The stacking strategy could effectively integrate the information which was extracted from CT and PET images by the SE-ResNet. The stacking model showed the potential to help clinicians making decision automatically and non-invasively by identifying suitable advanced patients with lung adenocarcinoma for EGFR‐TKI therapy.

## Data Availability Statement

The data sets analyzed during the current study are not publicly available for patient privacy purposes but are available from the corresponding author on reasonable request.

## Ethics Statement

The studies involving human participants were reviewed and approved by Tianjin Medical University Cancer Hospital Institutional Ethics Committee. The patients/participants provided their written informed consent to participate in this study.

## Author Contributions

DD, WX, and GY together designed the study. GY programmed the deep-learning based model and wrote the manuscript. ZW prepared the data samples and conducted research on CNN. YS conducted the statistical analysis. XL, YC, LZ, and QS collected the patient images, made the doctor diagnosis, conducted the pathology analysis, and performed image segmentation. WX also critically reviewed the manuscript. All authors contributed to the article and approved the submitted version.

## Funding

This work was supported by grants from the National Natural Science Foundation of China (Grant Nos. 81601377, 81501984, and 2018ZX09201015) and the Tianjin Natural Science Fund (Grant Nos. 16JCZDJC35200, 17JCYBJC25100, 18PTZWHZ00100, and H2018206600).

## Conflict of Interest

The authors declare that the research was conducted in the absence of any commercial or financial relationships that could be construed as a potential conflict of interest.

## Abbreviations

SE_CT_, Squeeze-and-Excitation Residual Network trained with CT images; SE_PET_, Squeeze-and-Excitation Residual Network trained with PET images; Stack_PET-CT_, the model integrating the results of Squeeze-and-Excitation Residual Network trained with CT images and Squeeze-and-Excitation Residual Network trained with PET images in decision level; Stack_PET-CT-Clinical_, the model integrating the results of clinical model, Squeeze-and-Excitation Residual Network trained with CT images and Squeeze-and-Excitation Residual Network trained with PET images in decision level.
