# Corrigendum: Prediction of EGFR Mutation Status Based on ^18^F-FDG PET/CT Imaging Using Deep Learning-Based Model in Lung Adenocarcinoma

**DOI:** 10.3389/fonc.2021.747316

**Published:** 2021-09-07

**Authors:** Guotao Yin, Ziyang Wang, Yingchao Song, Xiaofeng Li, Yiwen Chen, Lei Zhu, Qian Su, Dong Dai, Wengui Xu

**Affiliations:** ^1^Department of Molecular Imaging and Nuclear Medicine, Tianjin Medical University Cancer Institute and Hospital, National Clinical Research Center for Cancer, Tianjin Key Laboratory of Cancer Prevention and Therapy, Tianjin’s Clinical Research Center for China, Tianjin, China; ^2^School of Medical Imaging and Tianjin Key Laboratory of Functional Imaging, Tianjin Medical University, Tianjin, China

**Keywords:** adenocarcinoma of lung, fluorodeoxyglucose F18, positron emission tomography computed tomography, deep learning, epidermal growth factor receptor

In the original article, there was a mistake in **Table 2** as published. The clinical model was changed in the process of revising the manuscript. Due to our negligence, the AUC, sensitivity, and specificity of the clinical model for the training dataset were not correctly revised. The corrected **Table 2** appears below.

**Table 2:** Predictive performance of different models in the training dataset.

	AUC (95% CI)	Sensitivity (%)	Specificity (%)	Accuracy (%)
Stack_PET-CT_	**0.86 (0.80-0.91)**	71.75	**84.38**	**75.25**
SE_CT_	0.74 (0.67-0.80)	82.35	53.12	67.17
SE_PET_	0.75 (0.69-0.81)	**86.25**	56.25	72.22
Clinical model	0.63 (0.55-0.69)	50.98	71.88	60.10

The bold values represented the highest one of the evaluation indices.

The authors apologize for this error and state that this does not change the scientific conclusions of the article in any way. The original article has been updated.

## Publisher’s Note

All claims expressed in this article are solely those of the authors and do not necessarily represent those of their affiliated organizations, or those of the publisher, the editors and the reviewers. Any product that may be evaluated in this article, or claim that may be made by its manufacturer, is not guaranteed or endorsed by the publisher.

